# Danish Sonographers' Experiences of the Introduction of “Moderate Risk” in Prenatal Screening for Down Syndrome

**DOI:** 10.1155/2018/1646035

**Published:** 2018-10-09

**Authors:** Anne Møller, Ida Vogel, Olav Bjørn Petersen, Stina Lou

**Affiliations:** ^1^Department of Public Health, Aarhus University, Aarhus, Denmark; ^2^Center for Fetal Diagnostics, Aarhus University Hospital, Aarhus, Denmark; ^3^Department of Clinical Genetics, Aarhus University Hospital, Aarhus, Denmark; ^4^Fetal Medicine Unit, Department of Obstetrics and Gynaecology, Aarhus University Hospital, Aarhus, Denmark; ^5^DEFACTUM, Public Health & Health Services Research, Central Denmark Region, Aarhus, Denmark

## Abstract

**Objective:**

The aim of the study was to determine sonographers' experiences with the introduction of an offer of noninvasive prenatal testing (NIPT) to a new moderate-risk (MR) group at the combined first-trimester prenatal screening (cFTS).

**Study Design:**

A qualitative approach consisting of seven semistructured interviews with five sonographers (midwives and nurses). Data was analyzed using thematic analysis.

**Main Outcome Measures:**

Sonographers' perception of offering NIPT to women in MR.

**Results:**

The sonographers understood NIPT as a positive development in prenatal screening due to a safe procedure and high detection rates for trisomies 13, 18, and 21. Prior to the introduction of MR, the sonographers were concerned about inducing worry in pregnant women in this new risk group. However, the pregnant women responded very positively, which the sonographers attributed to several factors such as the women's overall reason for participating in prenatal screening, the simplicity of the NIPT procedure, and the communicative strategies used by the sonographers. The strategies included all sonographers using the same words and explanations, emphasizing that statistics were in the women's favor, initiating the presentation of MR with a positive message, and downplaying the MR category.

**Conclusion:**

Sonographers' communicative strategies succeeded in limiting worry in pregnant women in MR. As such, the findings are valuable for health professionals, who are responsible for communicating about prenatal screening results and diagnostic options.

## 1. Introduction

Over the past few decades, prenatal screening for chromosomal abnormalities in the fetus has been introduced in most Western countries. Prenatal screening technologies (such as combined first-trimester screening (cFTS)) allow for the identification of high-risk pregnancies. These women are subsequently offered a diagnostic test. Until recently, only invasive diagnostic tests involving a procedure-related risk of miscarriage were available (chorionic villus sampling (CVS) and amniocentesis). Recent studies have shown this risk of miscarriage to be as low as 0.1-0.2% [1]; however, it nevertheless remains a main concern amongst many high-risk pregnant women. Therefore, the noninvasive prenatal testing (NIPT) has potential to fundamentally change the current framework of prenatal care and diagnosis. NIPT is performed on maternal blood and is thus noninvasive and risk-free. It is not a diagnostic test, and abnormal test results must be verified by invasive testing. NIPT has, however, high accuracy in detecting trisomies 13, 18, and 21 and is generally viewed as a positive advancement in prenatal screening by pregnant women and clinicians [2].

NIPT has been introduced differently in different countries. In the Netherlands, NIPT was recently introduced as an alternative to cFTS, and, in countries like Denmark and Sweden, NIPT is offered as an alternative to invasive testing in high-risk groups [3]. Moreover, in many countries, private providers now offer NIPT to pregnant women who were previously considered low risk. In Denmark, NIPT has been tested as an offer to pregnant women with a cFTS result just below the high-risk cut-off (>1:300). Hence, a new category of risk was created: moderate risk (MR) (cFTS result between 1:300 and 1:699), and the number of pregnancies categorized as "at risk" was consequently increased. The offer of NIPT to MR was introduced in an attempt to improve detection rates but also derived consequences for both pregnant women and the health professionals caring for them.

Several studies show that a high-risk prenatal screening result generates a significant increase in anxiety for pregnant women [4]. Studies suggest that it is falling into the high-risk category itself (more than the exact risk figure) that generates worry and anxiety in pregnant women [5–7]. Based on this, one could hypothesize that a MR screening result could generate similar increased anxiety in pregnant women, as the health of the baby is questioned and additional tests are offered. Additionally, studies have shown that prenatal screening is often considered a routine part of prenatal care and, consequently, pregnant women are often not prepared for abnormal results [6]. This situation places a great demand on the health professionals responsible for communicating the screening results, and previous studies indicate that this communication is of great importance for the subsequent coping methods of the patients [8, 9].

With the introduction of a new MR group, the importance of clinical risk communication is further highlighted. Although considerable research has been devoted to pregnant women's experiences with participating in prenatal screening, rather less attention has been paid to the health professionals, who perform the scans and inform pregnant women about the results and options available. Thus, the introduction of a new MR group serves as an interesting case to investigate the management of communication of risk. The aim of this study was to investigate how clinicians experience and manage the introduction of an offer of NIPT to a new MR group.

## 2. Methods

Qualitative interviews were conducted in order to explore sonographers' experiences with the introduction of an offer of NIPT to a new MR group [10, 11].

### 2.1. Setting

The research was conducted at a fetal medicine unit at Aarhus University Hospital in Denmark. Denmark has a comprehensive and free-for-all screening program including cFTS and second-trimester scans [12, 13]. All routine scans are performed by sonographers, who also handle routine pre- and posttest information and counselling, including the reporting of high-risk and MR screening results. The sonographers are nurses/midwives specially trained in fetal medicine and are all certified in ultrasound from the Fetal Medicine Foundation, London.

The offer of NIPT to MR was implemented in Central Denmark Region in September 2015 as an addition to the cFTS. In this setting, MR was defined as a cFTS result of 1:300-1:699. In January 2017, the offer of MR NIPT, as well as the MR category as such, was discontinued following new guidelines from the Danish Health Authority [14].

### 2.2. Data Collection

Semistructured interviews were conducted by AM during February and March 2017. The interviews lasted approximately 30 minutes and were digitally recorded, anonymized, and transcribed verbatim. Recruitment was characterized by a convenience sample of five sonographers, of which two were interviewed twice (for participant characteristics, see Table 1). The main themes of the interview guide were the moderate-risk group, risk communication, consequences for the pregnant women, and the discontinuation (for examples of the interview questions, see Table 2). After interviews with four sonographers, the interviews were transcribed and initial findings were discussed among the authors. As there was a high degree of consensus among the interviewees, the interview guide was revised and probing questions were added. Subsequently, an additional sonographer was interviewed and two sonographers were interviewed again. However, these interviews added very limited new information to the material and researchers consequently estimated that data saturation was met [15, 16].

### 2.3. Data Analysis

**Figure 1:**
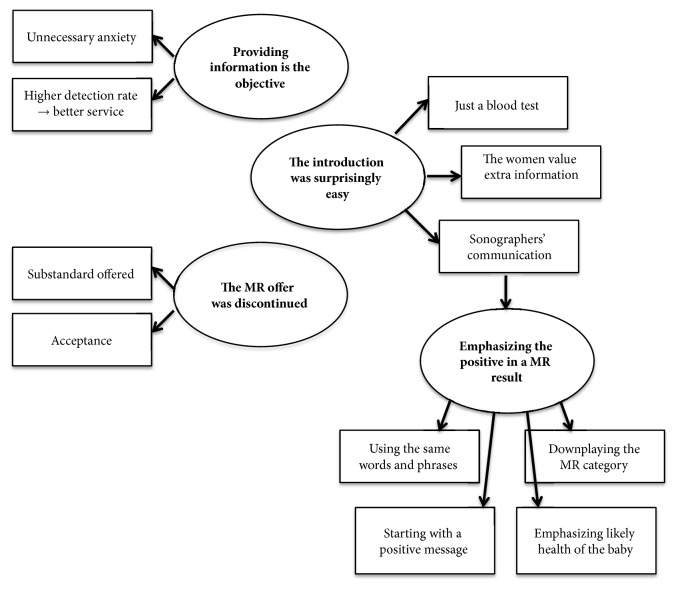
Thematic map.

Data was coded using NVivo 11.4 software (QSR International, Doncaster, Australia) and analyzed using thematic analysis, which is a theoretically flexible tool for analyzing qualitative data [17]. Data analysis was comprised of different steps. First, data familiarization was obtained by repeated reading of the transcripts. Second, the transcripts were coded into five main codes each with two to three subcodes. Third, the codes were clustered into themes by use of a thematic map to visualize the process. Fourth, reliability was ensured by reading through the coded data, which were compared to the themes. Finally, the themes were reviewed and named. Following this process, four themes were defined (see Figure 1).

### 2.4. Ethical Considerations

In Denmark, qualitative research does not require ethical approval from the National Committee on Health Research Ethics. Prior to the interviews, all sonographers at the fetal medicine unit received oral information about the study and informed consent was obtained. In the presentation of results, all participants have been carefully anonymized through the use of pseudonyms.

## 3. Results

The analysis resulted in the identification of four themes (see Figure 1) in the sonographers' experience with offering NIPT to MR: (1) Providing information is the objective. (2) Introduction was surprisingly easy. (3) Emphasizing the positive in a MR result. (4) The MR offer was discontinued.

### 3.1. Providing Information Is the Objective

Prior to the introduction of NIPT for MR, the sonographers were predominantly positive. NIPT was perceived as a positive technology that would allow for detection of more cases of Down syndrome:

I think that when you are in this [field] you want to offer the pregnant women as many answers as you can and as big a security as possible that the baby is healthy. In that way I think it is a good offer for them. (Emily)

This quote reflects what the interviewed sonographers perceived their professional objective to be: to provide as detailed and accurate information about the fetus as possible. Their aim was to provide sufficient information for the prospective parents either to feel reassured of the fetus's normal development or, in case of detecting something, to provide reliable information about potential risks and/or malformations and diagnostic options. According to the sonographers, NIPT was a tool for improving this provision of detailed and accurate information.

The sonographers expressed initial concerns towards the MR category:

There is a risk that we “medicalize” them. You know, to suddenly go from being normal to being “maybe-normal”. I had concerns about that. (Isabel)

The sonographers were cautious that the MR—also spoken of as a gray-zone result—would generate (in most cases unnecessary) increased anxiety in a larger group of women. The interviews reflected an effort among the sonographers to simultaneously “*tell it like it is*.” These concerns influenced the communicative strategies that the sonographers employed when the MR category was introduced.

In sum, the sonographers were positive towards implementing NIPT for MR. They were not concerned with whether the pregnant women chose NIPT or not but valued the possibility to provide them a choice and as much information as possible. However, the sonographers had initial concerns on the women's behalf seeing that it could cause unnecessary worry.

### 3.2. The Introduction Was Surprisingly Easy

The sonographers described the pregnant women's reactions to MR as “untroubled” and “surprisingly easy.”

They just accepted it and thought “well okay, then we have this [NIPT] offered”. But they did not get concerned or surprised or shocked. Not at all. (Tina)

In the sonographers' experience, the MR women perceived the offer of NIPT as an extra test and reassurance of the baby's health. Interestingly, none of the sonographers had experienced conflicts or negative reactions and offered the following explanations: First of all, the sonographers all accepted that pregnant women attended the cFTS in order to see their unborn child and share the experience with their partner. However, it was also the sonographers' experience that pregnant women were well aware of the medical purpose of the scan and came to the cFTS to gain information about their babies' health, and they all reported that many women preferred as much information as possible from them. The offer of NIPT fitted well with this purpose. Secondly, the noninvasiveness of NIPT meant that it did not put the pregnancy at risk. Compared to the invasive procedures, NIPT was simply a blood test:

[With the CVS] there is a risk - even though it is very, very low, it is different. And the other [NIPT] is just a blood test. I think, they [the pregnant women] often think; “it is probably not as serious”. (Naomi)

The sonographers argued that since most women have had a blood test taken, NIPT was an easier task for them to deal with. In comparison, an invasive test was more demanding for pregnant women, in terms of not only the risk of miscarriage, but also the discomfort of the invasive procedure, practical arrangements of getting extra days off, etc.

In sum, the pregnant women reacted surprisingly positively to MR and NIPT. The sonographers attributed this to the fact that the women were interested in information about their babies' health and the noninvasive, no-risk nature of NIPT. However, the sonographers assigned their own communication practice as the most important reason. This will be further described below.

### 3.3. Emphasizing the Positive in a MR Result

The sonographers were all attentive to the importance of appropriate clinical communication. Prior to the implementation of the MR, the sonographers had collectively discussed and agreed upon how to properly communicate the offer of NIPT to the MR group. One strategy was to ensure the collective use of approximately the same type of words and explanations in MR situations. This meant that the sonographers used collectively agreed on phrasing, examples, and figures of speech when communicating the MR result. This gave the sonographer a sense of security and speaking on behalf of her profession, not just her own experience and convictions. Another strategy was to emphasize that the fetus most likely was healthy. As one sonographer put it, this claim was fairly safe seeing that the risk was 1:300-1:699 and was earlier identified as low risk. Consequently, the sonographers felt confident enough using this strategy to not make the women feel unnecessarily worried. A third strategy was to avoid using the words* moderate risk* in the initial presentation of the screening result. The sonographers explained that, in their experience, the word* risk* had the power to trigger concern and worry in most pregnant women. Hence, they first underscored that the woman was* not* high risk, and the MR result was downplayed by referring to the offer of NIPT as something “extra” or “supplementary”:

I used this phrase for a couple of months: “if you had come in 2 months ago […] you would not have been offered anything, then you would just have been told that everything was fine and normal and no more tests, but now there is this, which is a good offer with a relatively little intervention.” (Tina)

Thus, the sonographers emphasized NIPT as something the MR group were fortunate to be offered compared to earlier times.

In sum, with the introduction of NIPT for MR, the sonographers made use of the following communicative strategies: collectively using words and explanations, emphasizing the probability of a normal result, initiating the conversations with a positive message, and downplaying the MR category.

### 3.4. The MR Offer Was Discontinued

Shortly before the interviews were conducted, the offer of NIPT to women in MR was unexpectedly discontinued. The sonographers expressed some concern with not being able to offer NIPT to this group of pregnant women:

[Phasing out NIPT] is as if you all of a sudden had to stop measure their heads or something like that. (Naomi)

This sonographer expressed a concern with delivering high-quality prenatal screening when no longer able to identify a MR group and offer NIPT. In her view, the discontinuation of MR increased the uncertainty for both the pregnant women and the sonographer herself. In principle, this made it more likely for the sonographers to overlook cases of Down syndrome. One sonographer explained her reaction when calculating a risk of 1:300-1:699 after the discontinuation:

Then I think “oh”, which was not a problem before. But it is because we want to take good care of them [the pregnant women]. (Laura)

The awareness of the former MR group made sonographers even more observant of risk results just above the high-risk cut-off. When asked if a woman's risk of giving birth to a Down syndrome baby was bigger after the discontinuation, one sonographer stated:

I have to say that I have not. If I start to go down that road I cannot be in this. So I have to be confident that this is normal. […] I have to cope with scanning for many years to come and be able to stand being in it mentally and not lie awake at night [wondering] if I did it well enough. (Isabel)

The sonographers expressed an intentional decision to accept the MR discontinuation as the right decision and they had collectively convinced themselves that they could still offer the same level of information (and reliability) in their scans. These decisions were reported to be necessary in order to be able to perform their daily work with confidence and satisfaction. Also, they did not want to contaminate the pregnant women with their own hesitance towards the discontinuation. One way to cope with this was to emphasize that they were not accountable for the decision to discontinue the MR group. They accepted it and perceived the argumentation to be fair. They agreed that it was vital for the number of cases detected to be in accordance with the financial expenses of the test.

In sum, the sonographers accepted the discontinuation of the MR group, despite it not being consistent with their wish to provide pregnant women with as much information about their babies' health as possible.

## 4. Discussion

Based on qualitative methods, this study aimed to investigate how sonographers experienced and managed the introduction of an offer of NIPT to a new MR group. The results show that in the sonographers' experience the pregnant women did not become particularly worried following a MR screening result. This untroubled response was largely attributed to the sonographers' communicative strategies: collectively using specific words and explanations, emphasizing that statistics were in the women's favor, initiating conversations with a positive message, and toning down the risk category. The sonographers were positive towards offering the new MR group NIPT, since it was consistent with their professional objective of providing detailed and high-quality information about the fetus to the pregnant women. Some sonographers found the discontinuation of the MR difficult, but they accepted the new guideline. The findings add new perspectives regarding how sonographers manage their daily work and regarding the significance of sonographers' communication within prenatal screening.

The results show that women with MR did not question the new risk category. This resonates with theories of modern society as a “risk society” where many health-related risks are invisible threats, whose presence is calculated through statistics and epidemiology [18]. Contrary to bodily symptoms, such as bleedings or pain, pregnant women cannot feel or observe symptoms of carrying a fetus with Down syndrome. Instead, the risk is calculated by computers and communicated by sonographers. Pregnant women rely on expert knowledge in order to identify and manage a number of invisible threats to the pregnancy and the fetus [19], and this context may help explain the ready acceptance of the MR category by the women in the study. Moreover, MR must be understood in the context of the Danish prenatal screening program that has a very consistent and high uptake (>90%) [20]. Studies have shown that Danish women are generally knowledgeable about and positive towards prenatal screening [21, 22], which means that the sonographers were communicating the MR to a—generally—proscreening and prochoice population, which may be another explanation why the MR result was so readily accepted. Moreover, the sonographers in this study were responsible for all risk communication without summoning a fetal medicine specialist, which may have downplayed and normalized MR women's experience of the situation.

The results show how the potentially worrisome and "invisible threat" to the baby's health—MR—was downplayed by the sonographers to “just” being a blood test and a current extra offer. A central premise in prenatal screening is neutral information and autonomous choice [23], and, consequently, one concern could be the potential undermining of pregnant women's autonomous choices by such communicative practices. Are pregnant women making informed choices, when the sonographers present NIPT as “just” a blood test? Health professionals play an important part in pregnant women's decision-making process [24]. However, studies have shown that, in order for women to make meaningful choices, more than neutral information about cut-off values and detection rates is needed [25]. Pregnant women also request experiential knowledge [26], alternative interpretations [7], and empathetic and collaborative communication [27]. Accepting these elements as central to good clinical communication, one must also take into account the specific situation that Danish pregnant women in MR are in: they have made an informed consent about participating in screening, they are NOT in high risk, and the test offered is risk-free. Thus, the context also supports a choice of NIPT. However, clinicians must always be careful and attentive to potential bias in their communication with patients, particularly to allow the less-likely choices to be made [21].

Ultimately, the discontinuation of MR put the sonographers in a position where they considered themselves unable to provide the same level of quality in their work. However, from the results, we can see how chosen standards (cut-offs, guidelines, and specific markers) are accepted and executed in order for clinical practice to function. Just like pregnant women trust the experts, the sonographers must trust the current guideline or protocol (MR or not) and act in accordance with it. This trust helps sonographers to navigate through gray zones (e.g., a “MR” result after the discontinuation) and to secure equal treatment for all patients. Thus, responsibility is delegated to the current cut-off, as the authoritative designator of high risk and low risk, around which the sonographers arrange their work and communication.

Consequently, the results point to two central motivations that underpin the sonographers' daily clinical work and communication with pregnant women: First, sonographers expressed a personal concern and responsibility for examining the fetus thoroughly and detecting as many cases of Down syndrome (and other abnormalities) as possible. Importantly, the sonographers were not concerned with whether the women chose NIPT or not, but with providing information and the possibility of choice. Secondly, the sonographers organized their work and their communication to ensure that pregnant women did not become unnecessarily worried by the information provided by ultrasound, including information about MR. Based on this study, it must be concluded that the sonographers—in their understanding—succeeded in balancing these paradigms in the implementation of NIPT to pregnant women with MR.

### 4.1. Limitations

The results are based on a small sample of sonographers recruited from an ultrasound clinic in Denmark, which limits generalizability of results. First, Danish sonographers are educated nurses and midwives and thus have expertise in clinical communication. Additionally, the sample of sonographers is small and relatively experienced, which may influence the results. Furthermore, there is a high uptake in cFTSs among Danish women, which may reflect a rather positive attitude towards screening compared to other countries. Finally, this study has focused on the sonographers' experiences and their impression of pregnant women's response to MR. Future research should include an investigation of pregnant women's own experiences.

## 5. Conclusion

This study is a step towards understanding how sonographers manage the introduction of a new technology (NIPT) and a new risk category (MR). The sonographers used different collective strategies to communicate a MR risk result. Overall, the sonographers experienced the pregnant women responding positively to the MR NIPT offer and they did not detect an increase in worry among the MR group. However, further research is needed to explore pregnant women's experiences of being categorized as MR, their assessment of the clinical communication, and their reasons for choosing/declining NIPT.

## Figures and Tables

**Table 1:** Participant characteristics.

**Name**	**Age**	**Education**	**Years of work experience**
Isabel	50	Nurse	11
Emily	50	Midwife	12
Laura	46	Nurse	12
Tina	44	Nurse	12
Naomi	32	Nurse	2

**Table 2:** Topic guide.

**Topic**	**Examples of questions**
Implementation of NIPT	(i) Do you think implementing NIPT was a good idea? (Why/why not?)
	(ii) How was the pregnant women's reaction to NIPT?

The moderate-risk group	(i) Do you think there were any problems connected to the fact that pregnant women could be identified as in moderate risk? (Any benefits?)
	(ii) Did you think differently about the risk of the pregnant women that were in a moderate risk group compared to earlier times? (Why/why not?)

Risk communication	(i) Do you think about the word risk when you talk to the pregnant women?
	(ii) How exactly did you tell pregnant women that they were in a moderate risk group?

Consequences for the pregnant women	(i) How did the women react being identified as in moderate risk? (Examples?)
	(ii) Did you have a feeling of causing the women unnecessary anxiety?

The discontinuation	(i) How do you feel about that the moderate risk group no longer exists?
	(ii) What thoughts do you have about that pregnant women, who earlier would be in a moderate risk group and offered NIPT, today not are offered any additional tests?

## Data Availability

The authors agree to make the data underlying the findings of the study available upon reasonable request and for the purpose of academic, noncommercial research. Data are available from the Center for Fetal Diagnostics for researchers who meet the criteria for access to confidential data.
